# Comparing Standardized and Parent-Reported Motor Outcomes of Extremely Preterm Infants

**DOI:** 10.3390/children6080090

**Published:** 2019-08-01

**Authors:** Maeve Morgan-Feir, Andrea Abbott, Anne Synnes, Dianne Creighton, Thevanisha Pillay, Jill G. Zwicker

**Affiliations:** 1 Department of Occupational Science, Occupational Therapy, University of British Columbia, Vancouver, BC V6T 2B5, Canada; 2 Department of Pediatrics, University of British Columbia, Vancouver, BC V6H 3V4, Canada; 3 Brain, Behaviour, Development Theme, BC Children’s Hospital Research Institute, Vancouver, BC V5Z 4H4, Canada; 4 Neonatal Follow-Up Program, BC Women’s Hospital, Vancouver V6H 4J4, Canada; 5 Department of Pediatrics, Cumming School of Medicine, University of Calgary, Calgary, AB T2N 4N1, Canada; 6 Division of Family Practice, Victoria General Hospital, Victoria, BC V8Z 6R5, Canada; 7 Neuromotor Program, Sunny Hill Health Centre for Children, Vancouver, Canada

**Keywords:** motor outcomes, extremely preterm infants, Bayley-III, Ages and Stages Questionnaire

## Abstract

Extremely preterm infants are at increased risk of motor impairment. The Canadian Neonatal Follow-Up Network (CNFUN) afforded an opportunity to study the outcomes of extremely preterm children. The purpose of this study was to compare 18-month corrected age (CA) motor outcomes of extremely preterm infants with parent-reported functional outcomes at 3 years CA. CNFUN data of 1376 infants were used to conduct chi-square analyses to compare Bayley-III motor scores (composite, gross, and fine motor) at 18 months CA with parent-reported Ages and Stages Questionnaire motor scores (gross and fine motor) at 3 years CA. The correlation of motor scores at 18-months CA with parent-reported gross and fine motor scores at 3 years CA was also examined. We found that 1 in 5 infants scoring within or above the average range on the Bayley-III had parent-reported functional fine and gross motor difficulties at 3 years CA. Bayley-III scores were only moderately correlated with functional motor outcomes. Results of the study suggest that the Bayley-III at 18 months CA was able to detect the majority of infants with motor problems, but not all; therefore, ongoing follow-up of extremely preterm infants is required. The Bayley-III motor composite score has greater clinical utility compared to sub-scale scores.

## 1. Introduction

Extremely preterm infants are at high risk for adverse neurodevelopmental outcomes such as visual, hearing, language, motor, and cognitive impairments that emerge in early childhood, requiring regimented follow-up and assessment [1,2]. Motor impairments in children born extremely preterm include cerebral palsy and developmental coordination disorder [3,4]. In Canada, children who are born extremely preterm are referred to neonatal follow-up clinics that aim to investigate the effects of neonatal practices, detect impairments early, promote early intervention, and provide training to healthcare professionals. Although practices vary between neonatal follow-up programs [5], most children born extremely preterm are eligible for a standardized neurodevelopmental assessment at 18 months corrected age (CA).

For children born between September 2009 and April 2011, the Canadian Neonatal Follow-up Network (CNFUN) collected longitudinal information including the Bayley Scales of Infant and Toddler Development III (Bayley-III) at 18 months CA and a parent- or caregiver-completed questionnaire at 3 years CA for extremely preterm infants born <29 weeks gestational age [6]. With 26 contributing sites in Canada, the CNFUN provides a unique opportunity to study the motor outcomes of extremely preterm children in a large national sample.

The Bayley-III is a widely accepted assessment of neurodevelopment that investigates motor, language, and cognitive capabilities [7]. The Bayley-III became the reference standard for investigations of motor outcomes when it was introduced in 2006 with strengthened psychometric qualities [7,8,9,10]. However, evidence has shown that the Bayley-III, including the motor composite score, underestimates impairment compared to previous editions. [11,12]. More research is required to determine which threshold should be used for impairment for the motor composite score of the Bayley-III [10,12]. Typically, scores below one standard deviation (- 1SD) from the normative mean indicate mild impairment and scores below 2 standard deviations (- 2SD) indicate moderate-to-severe impairment.

Parent-report measures, such as the Ages and Stages Questionnaire, Third Edition (ASQ-3), are increasingly used as an assessment of function [13,14]. When used as a screening tool, parent report measures can reduce the need for a clinical assessment of preterm infants [15,16]. Previous research on moderate to late preterm infants has found good concurrent validity between the Bayley-III and the Parent Report of Children’s Abilities-Revised, suggesting a relationship between standardized and parental report measures [16]. There remains a gap in the literature, however, regarding parent-reported motor outcomes for extremely preterm infants.

Using a large national cohort, the aims of the current study were to investigate: (1) the proportion of extremely preterm infants scoring within and above the average range of the motor component of Bayley-III at 18 months CA and in the monitoring zone (− 1SD) or the abnormal zone (− 2SD) on the ASQ-3 at 3 years CA; (2) the correlation between Bayley-III motor composite and individual fine and gross motor subscales with fine and gross scales of the ASQ-3; and (3) the predictive validity of Bayley-III motor composite and gross and fine motor subscales at 18 months CA with the ASQ-3 motor scores at 3 years.

## 2. Materials and Methods

### 2.1. Study Design

We conducted a retrospective analysis of the prospective CNFUN cohort study. All 26 Canadian neonatal follow-up programs in Canada participated in CNFUN as part of the Canadian Institute of Health Research Maternal Infant Care study, with research ethics board approval obtained at all participating sites. The legal guardians of the children included in the study consented to the collection and sharing of data by the CNFUN when required by the local research ethics boards. The Declaration of Helsinki was used as an ethical guide for completion of the study. This study was reviewed by the CNFUN Steering Committee and approved by the University of British Columbia and British Columbia Children’s and the Women’s Hospital Research Ethics Board.

### 2.2. Study Participants

**Figure 1:**
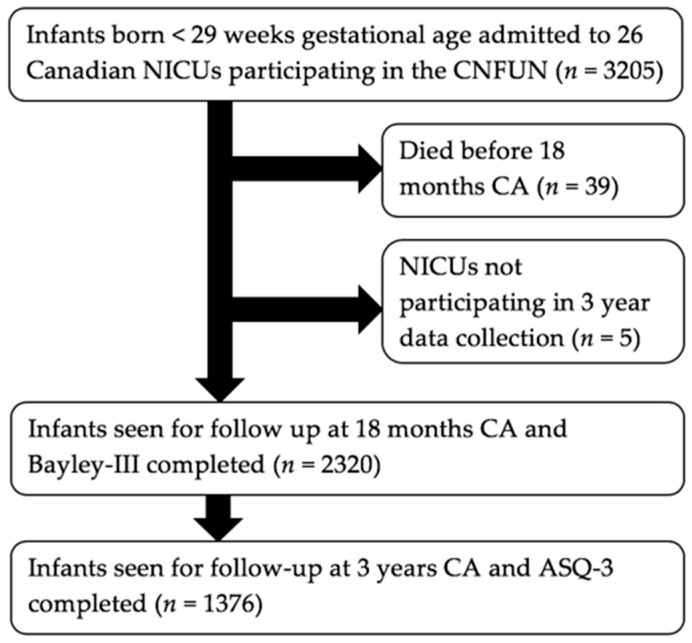
Flow chart depicting study participant data.

The participants were selected from 3205 extremely preterm infants less than 29 weeks gestational age, born between 1 April 2009 and 30 September 2011 and admitted to one or more of the participating Neonatal Intensive Care Units (NICUs) affiliated with a CNFUN site. All families were eligible regardless of the language spoken, comorbidities, location, or involvement in other programs. Of the 3205 infants eligible for inclusion in the study, 1376 parents/legal guardians completed the 33-, 36-, or 42-month version of the ASQ-3 (Figure 1).

### 2.3. Data Collection Protocol

This study used data collected at the 21 sites that were able to participate in both the 18-month and the 3-year CNFUN data collection.

Bayley-III test scores were calculated at each site according to the instructions in the Bayley-III manual. The Bayley-III motor composite has a mean of 100 and SD of 15, while the gross and fine motor standard scores have a mean of 10 and an SD of 3. As such, Bayley-III motor composite cut-offs of one standard deviation (<85) and two standard deviations (<70) below the normative mean were used to classify scores in the range of mild impairment and moderate-to-severe impairment, respectively. Similarly, Bayley-III gross and fine motor scaled score cut-offs of one standard deviation (<7) and two standard deviations (<4) below the normative mean were used. The ASQ-3 was scored centrally at the CNFUN coordinating site and was interpreted by investigators as per the ASQ-3 manual. The scores were defined as falling in the normal range, monitoring zone (− 1SD), or abnormal range (− 2SD).

### 2.4. Statistical Analyses

Descriptive data analysis (medians, inter-quartile ranges (IQR), and percentages) regarding birth data (sex, gestational age, birth weight), 18-month CA motor outcomes from the Bayley-III, and 3-year CA motor outcomes from the ASQ-3 was conducted. Chi-square analyses were used to compare the proportion of children with motor impairments on the Bayley-III scales at 18 months (motor composite, fine motor, gross motor) with the proportion of children with reported motor difficulties on the ASQ-3 at 3 years CA. We also examined the relationship between Bayley-III and ASQ-3 scores using Pearson correlation coefficients. To assess the predictive ability of two Bayley-III motor composite cut-off points (<70 and <85) on abnormal or borderline ASQ-3 gross or fine motor outcomes, a series of sensitivity and specificity calculations were performed using 2 × 2 chi-square analyses, with the monitoring zone and abnormal range on the ASQ-3 grouped together as “poor outcome”. As a post hoc analysis, we also examined the sensitivity and specificity at different cut-off scores.

## 3. Results

The clinical characteristics of the 1376 infants included in the study are shown in Table 1. Infants who were lost to follow-up at 3 years were of a similar gestational age (median: 27 weeks; IQR: 26, 28; *p* = 0.09) but of a higher birthweight (median: 945 grams; IQR: 780, 1107; *p* = 0.04) compared to the infants who were seen at a 3-year follow-up.

Most of the infants scored within the average range (≥85) on the Bayley-III motor composite at 18-months CA. Most of these infants had no reported motor difficulties at 3 years; however, 22% fell within the abnormal or monitoring zone for gross motor skills (Table 2) and 20% fell within the abnormal or monitoring zone for fine motor skills on the ASQ-3 (Table 3). Similarly, the majority of the infants who scored within or above the average range (≥7) on both the Bayley-III gross and the fine motor subscales had no parent-reported gross or fine motor difficulties at 3 years CA (Table 4; Table 5). However, 22% of these infants fell within the abnormal or monitoring zone for fine and gross motor difficulties on the ASQ-3 (Table 4 and Table 5).

While our primary question was related to examining the number of infants scoring within the average range on the Bayley-III at 18 months CA who experienced functional motor difficulties later in childhood (false negatives), it is also interesting to note how many children who scored below average on the Bayley-III did not have functional difficulties at 3 years of age (false positives). Of the children who scored in the normal range on the ASQ-3 gross motor scores, only 17/850 (2%) had a motor composite score ≤70 on the Bayley-III (Table 2). Similarly, 33/899 children (4%) with normal ASQ-3 fine motor scores scored ≤70 on the Bayley-III (Table 3).

Strong correlations were found between both the Bayley-III motor composite and the ASQ-3 gross motor outcomes (*r* = 0.51) and the Bayley-III gross motor scale and the ASQ-3 gross motor outcomes (*r* = 0.52) (both *p* < 0.01). Moderate correlations were found between both the Bayley-III motor composite and the ASQ-3 fine motor outcomes (*r* = 0.40) and the Bayley-III fine motor scale and the ASQ-3 fine motor outcomes (*r* = 0.43) (both *p* < 0.01).

For the Bayley-III motor composite score cut-off <85, sensitivity for the prediction of ASQ-3 gross motor outcomes was 45% and specificity was 88%, while sensitivity for the prediction of ASQ-3 fine motor outcomes was 40% and specificity was 84%. Comparatively, sensitivity was lower and specificity was higher using a cut off <70 on the Bayley-III motor composite, with the 23% sensitivity and 98% specificity for the ASQ-3 gross motor outcomes, and 18% sensitivity and 96% specificity for the ASQ-3 fine motor outcomes. Sensitivity and specificity for alternate cut-off scores are indicated in Table 6.

## 4. Discussion

### 4.1. Bayley-III Scores at 18 Months CA and ASQ Scores at 3 Years CA

The results of the study indicate that the majority of children that achieve average and higher scores on the motor composite or individual fine and gross motor subscales of the Bayley-III at 18-months CA went on to achieve normal scores on the ASQ-3 gross and fine motor subscales at 3 years CA. However, at least 20% of extremely preterm infants scoring average or higher on the Bayley-III motor scales at 18 months CA achieved an outcome in the monitoring or abnormal zone of the ASQ-3 at 3 years. In other words, one in five infants that scored average or higher on the Bayley-III at 18-months CA experienced parent-perceived functional motor difficulties at 3 years CA.

Current follow-up intervention practice includes assessment of children during infancy and at 18 months CA to determine early intervention practices and potentially discharge from follow-up. These data, however, suggest that 18 months CA may be too early to draw any definitive conclusions about a child’s motor development; approximately one in five children achieving abnormal results on the ASQ-3 at 3 years CA scored within or above the normal range at 18 months CA on standardized testing. Previous research also suggests that, when used as a screening tool, parent report measures can reduce the need for clinical assessment of preterm infants [15,16]. While increased and extended standardized assessment of extremely preterm infants may represent a cost to the healthcare system, this suggests that increased parent-reported assessment may pose a low-cost alternative allowing for extended follow-up.

### 4.2. Correlation of Bayley-III Motor Scores at 18 Months CA and ASQ Motor Scores at 3 Years CA

A strong, statistically significant correlation was found between the Bayley-III motor composite score or gross motor subscale at 18-months and ASQ-3 gross motor outcomes at 3 years. Comparably, the correlation of the Bayley-III motor composite or fine motor subscale at 18-months with ASQ-3 fine motor outcomes at 3 years was only moderate. The difference in these correlations may be due to the later emergence of fine motor skill and the associated difficulty of assessing fine motor ability in young children [9]. In addition, in terms of the parent report on the ASQ-3, it may also be more difficult for parents to notice the quality of their child’s fine motor skills (e.g., drawing a vertical or horizontal line, copying a circle, stringing beads, using scissors, how he/she holds a crayon) compared to their child’s gross motor skills (e.g., standing on one foot, jumping, hopping, kicking or throwing a ball).

### 4.3. Predictive Validity of Bayley-III Motor Scores at 18 Months CA to ASQ Motor SCORES at 3 years CA

For all of the cut-off scores analyzed, the Bayley-III showed stronger specificity compared to sensitivity. This indicates a stronger ability to accurately predict extremely preterm infants who would go on to score within the normal zone of the ASQ-3 at 3 years. Sensitivity was low, indicating poor ability of the Bayley-III to accurately identify extremely preterm infants who experienced motor difficulties at 3 years. This contributes to the growing body of research suggesting that they Bayley-III may underestimate disability compared to previous versions [10,12].

Sensitivity was higher with the cut-off score of <85 on the Bayley-III but was also related to lower specificity scores. Further research is required to determine which cut-off score on the Bayley-III motor composite more accurately predicts motor outcomes. Preliminary evidence suggests that a motor composite cut-off point of <73 may improve sensitivity and specificity values [10]. In our study, a score of <73 also showed greater specificity than higher cut-off scores, but <85 had the greatest specificity.

### 4.4. Study Limitations

This retrospective CNFUN cohort study has some limitations. Firstly, inclusion of a comparison group was not possible. A comparison group would have allowed for analysis of normative outcomes, leading to a better knowledge of over- or under-estimation of disability by the measures used in the study. Our study is limited in comparing results of standardized and parent-reported measures at two different points in time; having a standardized motor assessment at 3 years would have further validated these results. While the purpose of Bayley-III and ASQ-3 is very different (e.g., diagnosis versus screening), this study provides important information regarding the predictive validity to motor outcomes from the parents’ perspective. A significant limitation is that intervention data between 18 months and 3 years CA were not systematically collected across all sites. As such, we were not able to adjust for the effects of intervention on our results; however, intervention would be expected to reduce the proportion of children with functional motor difficulties at 3 years. Not all eligible caregivers completed the ASQ-3, leading to attrition bias. We did not account for socio-economic status (SES) in our analysis; while we expect that SES would remain constant within families across the two time points of evaluation, differences between families may account for the variability in scores. Finally, while the sample size of the study was large, the results cannot be generalized to all infants born extremely preterm, especially outside of Canada.

## 5. Conclusions

The results of the study suggest that the Bayley-III at 18 months CA was able to detect the majority of infants with motor problems, but not all; therefore, ongoing follow-up of extremely preterm infants is required. The Bayley-III motor composite score had the strongest correlation with the ASQ-3 results, suggesting a greater clinical utility of the Bayley-III motor composite score compared to fine and gross motor sub-scale scores. Although further research is required to determine which cut-off score on the Bayley-III motor composite more accurately predicts motor outcomes, specificity was higher than sensitivity. These results may inform early intervention practices, including the length and method of neonatal follow-up. Future research is needed to investigate use of parent-reported measures as a low-cost avenue for extended follow-up services.

## Figures and Tables

**Table 1:** Clinical Characteristics.

**Birth Data**	**Median (IQR) or N (%)**
Male	741 (54)
Gestational age (weeks)	27 (25, 28)
Birth weight (grams)	910 (770, 1090)
Multiple births	387 (28)
**18 Month CA Outcomes**	**Mean (SD) or N (%)**
Motor Composite Score	
Mean	91.5 (15)
≥85	1002 (73)
70–84	198 (14)
<70	92 (7)
Not performed (untestable)	84 (6)
Definite Cerebral Palsy	78 (6)
Gross Motor Subscale	
Mean	7.7 (3)
≥7	946 (69)
4–6	249 (18)
<4	104 (7)
Not performed (untestable)	77 (6)
Fine Motor Subscale	
Mean	9.6 (3)
≥7	1141 (83)
4–6	121 (9)
<4	38 (3)
Not performed (untestable)	76 (5)
**3-year CA Outcomes**	**Mean (SD) or N (%)**
Gross Motor	
Mean	48.70 (14)
Monitoring Zone (−1SD)	184 (14)
Cut off Zone (−2SD)	235 (18)
Fine Motor	
Mean	40.97 (17)
Monitoring Zone (−1SD)	216 (16)
Cut off Zone (−2SD)	142 (11)

**Table 2:** Comparison of Bayley-III Motor Composite and ASQ Gross Motor (GM).

	ASQ GM Normal N (%)	ASQ GM Monitoring N (%)	ASQ GM Abnormal N (%)	*X*^2^	*p*-Value
Bayley Motor ≥85	745 (78)	126 (13)	89 (9)		
Bayley Motor 70–84	88 (46)	41 (21)	63 (33)	285.2	<0.01
Bayley Motor <70	17 (19)	8 (9)	65 (72)		

**Table 3:** Comparison of Bayley-III Motor Composite and ASQ Fine Motor (FM).

	ASQ FM Normal N (%)	ASQ FM Monitoring N (%)	ASQ FM Abnormal N (%)	*X*^2^	*p*-Value
Bayley Motor ≥85	751 (80)	131 (14)	59 (6)		
Bayley-Motor 70–84	115 (61)	43 (23)	30 (16)	139.4	<0.01
Bayley Motor <70	33 (37)	19 (21)	37 (42)		

**Table 4:** Comparison of Bayley-III Gross Motor subscale and ASQ Gross Motor (GM).

	ASQ GM Normal N (%)	ASQ GM Monitoring N (%)	ASQ GM Abnormal N (%)	*X*^2^	*p*-Value
Bayley Gross Motor ≥7	709 (78)	117 (13)	79 (9)		
Bayley Gross Motor 4–6	130 (54)	48 (20)	64 (26)	295.9	<0.01
Bayley Gross Motor <4	18 (17)	10 (10)	74 (73)		

**Table 5:** Comparison of Bayley-III Fine Motor subscale and ASQ Fine Motor (FM).

	ASQ FM Normal N (%)	ASQ FM Monitoring N (%)	ASQ FM Abnormal N (%)	*X*^2^	*p*-Value
Bayley Fine Motor ≥7	841 (78)	162 (15)	70 (7)		
Bayley Fine Motor 4–6	55 (48)	28 (24)	32 (28)	192.3	<0.01
Bayley Fine Motor <4	6 (16)	7 (19)	24 (65)		

**Table 6:** Sensitivity and Specificity of Bayley-III Motor Composite Scores at 18 months CA with Poor ASQ-3 Motor Outcomes (monitoring zone or abnormal range) at 3 years CA.

	ASQ-3 Gross Motor		ASQ-3 Fine Motor	
	Sensitivity (%)	Specificity (%)	Sensitivity (%)	Specificity (%)
Bayley Motor Composite <85	45	88	40	84
Bayley Motor Composite <84	45	88	40	84
Bayley Motor Composite <83	45	88	40	84
Bayley Motor Composite <82	38	92	34	88
Bayley Motor Composite <81	38	92	34	88
Bayley Motor Composite <80	38	92	34	88
Bayley Motor Composite <79	30	95	27	92
Bayley Motor Composite <78	30	95	27	92
Bayley Motor Composite <77	30	95	27	92
Bayley Motor Composite <76	26	96	25	94
Bayley Motor Composite <75	26	96	25	94
Bayley Motor Composite <74	26	96	25	94
Bayley Motor Composite <73	23	98	21	95
Bayley Motor Composite <72	23	98	21	95
Bayley Motor Composite <71	23	98	21	95
Bayley Motor Composite <70	23	98	18	96
